# When control words lose control: emergence of top-down processing effects in Spanish spoken word recognition

**DOI:** 10.3389/fpsyg.2026.1832323

**Published:** 2026-09-03

**Authors:** Angela Swain, Melinda Fricke

**Affiliations:** 1 Department of Spanish, Italian and Portuguese, The Pennsylvania State University, University Park, PA, United States; 2 Department of Linguistics, University of Pittsburgh, Pittsburgh, PA, United States

**Keywords:** Andalusian Spanish, auditory lexical processing, language attitudes, Peninsular Spanish, sociophonetic variation

## Abstract

Research on cross-dialectal spoken word recognition suggests integrated processing of social and linguistic information, enabled by the encoding of fine-grained sociophonetic detail. Asymmetries in encoding strength have also been documented, especially concerning idealized or socially meaningful variants. To date, the literature has demonstrated discrepancies between immediate and long-term processing, the latter being understudied, along with an overwhelming focus on English varieties. The current study therefore examined immediate and long-term processing of three /s/ variants in Peninsular Spanish: [s̺], [s̪], and [s̪^θ^]. Each variant represents distinct geographic and social associations, providing a fruitful context to assess the implicit interaction of linguistic and extralinguistic information in processing. Seville capital participants (*N* = 42) completed a long-term form priming task containing word-initial /s/ items. Talkers (*N* = 6; one man and one woman per location) were from Seville capital, Seville outskirts, or central Spain. Response times initially demonstrated recognition equivalence in terms of processing speed: response times showed no differences among the three word-initial /s/ variants. However, faster processing of /s/ words produced by talkers from Seville capital and central Spain emerged after a long lag. Moreover, no recognition differences were observed between the two variants. Participants were less accurate in recognizing /s/ words produced with the Seville outskirts variant, suggesting processing difficulty. Unexpectedly, control words produced by one Seville capital talker were recognized especially quickly. The findings are discussed with respect to memory inequality and top-down processing effects and support the interaction of social and linguistic information in spoken word recognition.

## 1 Introduction

Research on variability in speech processing strives to detail the fundamental mechanisms responsible for facilitating spoken word recognition and encoding. Exemplar theories (e.g., Goldinger, 1998; Johnson, 1997) emphasize the storage of detailed acoustic episodes, also capturing social categories such as talker gender, dialect, and contextual information (e.g., Bybee and Torres Cacoullos, 2008; Pierrehumbert, 2016). Studies examining talker-specificity (i.e., the idea that listeners encode precise information about the voice that produced a given word) overwhelmingly support the retention of fine-grained acoustic detail (e.g., Palmeri et al., 1993). However, contemporary research has questioned the generalizability of such effects upon featuring more diverse talker voices (e.g., Clapp et al., 2023).

Research in cross-dialectal spoken word recognition has contributed more nuance to the emerging picture, with some results inspiring an update to models that solely prioritized frequency-based encoding. One discovery has been memory inequality (Sumner et al., 2014; Clapp et al., 2023), springing from results that showed better memory for a relatively less frequent variant (e.g., Sumner and Kataoka, 2013), also concerning the social relevance of local variants (e.g., Clopper et al., 2016; López Velarde and Simonet, 2019). Questions therefore persist about the mechanisms controlling exactly what is encoded and under what circumstances.

Contemporary work has additionally expanded stimuli from single words to whole sentences. Results from Clapp and Sumner (2025) suggested that listeners can also encode full sentences with striking acoustic detail. Moreover, as a distracted experimental condition facilitated stronger talker specificity effects, the authors hypothesized that acoustic encoding is implicit and influenced by allocation of attention. In another study, the implicit nature of encoding was explored by testing non-proficient Spanish speakers in California and Texas. Todd et al. (2023) discovered that habitual ambient exposure to Spanish facilitated the subconscious development of lexical knowledge, with participants’ attitudes towards local Spanish speakers modulating the strength of their knowledge.

Recent advances notwithstanding, several important gaps persist. First, the majority of studies exploring cross-dialectal spoken word recognition featured varieties of English (e.g., GA/NYC English, Sumner and Samuel, 2009). López Velarde and Simonet (2019) are an exception, analyzing [t͡ʃ]/[ʃ] variation in Mexican *norteño* Spanish. Testing other languages is vital to generalizations regarding memory storage in the lexicon. Another shortcoming is the lack of evaluative data collected regarding the talkers heard in experiments (Clopper, 2021). Since indexical information is encoded (Pierrehumbert, 2016), capturing participants’ language attitudes can further highlight interactions of linguistic and social information in processing.

To address these gaps, the current study explored spoken word recognition involving three /s/ variants in Peninsular Spanish, each associated with distinct geographic locations. North-central regions of Spain produce an apico-alveolar variant [s̺], cities in western Andalusia such as Seville are associated with a dental variant [s̪], and a variant with interdental mixing, [s̪^θ^], is frequent in many towns outside of Seville and coastal cities such as Cádiz (e.g., Penny, 2004; Regan, 2017). These variants also possess strong indexical information. North-central varieties, in which [s̺] is produced, are the national standard; they are widely represented in the media and associated with status, urbanness, and clarity (e.g., Santana Marrero, 2016–2017, 2018, 2022). Andalusian varieties, encompassing both [s̪] and [s̪^θ^], are innovative (e.g., Villena-Ponsoda, 2008). Speakers of Andalusian Spanish often neutralize their accents in formal settings, the media, and political spheres (e.g., Cruz Ortiz, 2020; Santana Marrero, 2022).

Though both [s̪] and [s̪^θ^] are Andalusian variants, they represent disparate social implications. The dental variant [s̪] indexes higher perceived socioeconomic status, formality, and urbanity compared to [s̪^θ^] (e.g., Regan, 2019, 2022). On the contrary, [s̪^θ^] is linked to lower perceived education and socioeconomic status, along with higher ruralness and perceived humor (e.g., Henriksen et al., 2023; Regan, 2019, 2022). Gender differences also emerge; women producing [s̪^θ^] received lower status ratings than men with the same variant (Regan, 2022).

The linguistic landscape in Seville further exemplifies the differential status of the two Andalusian /s/ variants via orthographic cases of *seseo* and *ceceo* (Pons Rodríguez, 2012).^1^ *Seseo* appeared in planned signage, but involuntary *ceceo* was absent, even in culturally specific settings such as the names of *casetas*, “tents” at the Seville fair (Pons Rodríguez, 2012, p. 247). While *seseo* holds regional prestige and broader acceptability, *ceceo* is socially stigmatized and restricted to informal speech domains (e.g., Méndez-Ga de Paredes and Amorós-Negre, 2018). Both *seseo* and *ceseo* coexist with frequent coda /s/ weakening, which is widespread across Andalusian varieties and less prevalent in north-central zones (e.g., Villena-Ponsoda, 2008).

Taken together, Seville capital natives have a three-way familiarity with the /s/ variants: they most frequently produce the dental variant [s̪], are geographically close to the interdental Andalusian variant [s̪^θ^], and are consistently exposed to [s̺] through the national media. To add further complexity, Seville capital speech is considered the norm in western Andalusia, its regional status directly competing with the national standard despite an expansion of north-central variants into southern speech (e.g., Hernández-Campoy and Villena-Ponsoda, 2009; Santana Marrero, 2022; Villena-Ponsoda and Ávila-Muñoz, 2014). This complex dynamic provides an optimal context to explore the processing of social and linguistic information, operationalized by three word-initial /s/ variants.

Participants in the current study completed a long-term form priming task; the results detailed below were part of a larger experiment (Swain, 2023). Immediately prior to the priming task, participants completed a Verbal Guise (Markel et al., 1967), answering dialect classification and evaluation questions for all talkers featured in the form-priming experiment. We presented the tasks in this order to familiarize participants with the talkers and facilitate activation of social information. Each talker produced word-initial /s/ tokens in the Verbal Guise, along with other phonetic traits that cue sociodemographic information (e.g., extensive coda /s/ weakening with Andalusian varieties).

The current study addresses the following research questions:

1. How successful are Seville capital listeners in recognizing Spanish nouns containing three word-initial /s/ variants in immediate processing?
2. How does introducing a long lag between prime and target presentation affect /s/ word recognition?
3. To what extent do previously activated social categories influence spoken word recognition?

## 2 Method

### 2.1 Participants

Participants (*N* = 42) were all born and raised in Seville capital or moved there during infancy.^2^ All participants reported Spanish as their dominant language and were at least 18. The participant pool was balanced across gender (*N* = 19 men, *N* = 23 women) and included a broad age range (*M* = 38.5 years, range = 18–64 years), featuring greater listener diversity to ensure better generalizability (e.g., Clapp et al., 2023). Education levels of participants also varied, with approximately half (*N* = 19) completing up to *Bachillerato* in Spain (i.e., upper-secondary education). The remaining participants had earned undergraduate (*N* = 16) or graduate (*N* = 7) degrees.

### 2.2 Stimuli

All stimuli were disyllabic items with penultimate stress (*N* = 720). Half of the items were legitimate Spanish words and half were pseudowords. All real words were nouns divided into three categories: word-initial /s/ items (e.g., *sangre*, “blood”), controls (e.g., *plata*, “silver”), and fillers (e.g., *reina*, “queen”). We used the Spanish lexical database *Español Palabras* (EsPAL; Duchon et al., 2013) to select the words, controlling for frequency and phonological neighborhood density. Controls and /s/ words all ended in vowels; most fillers also ended in vowels, though a variable coda was permitted across these items. Controls and /s/ words were also maximally constrained to avoid salient phonetic features in Peninsular Spanish. Pseudowords maintained Spanish phonological rules and were formed by selecting the best candidates generated in Wuggy (Keuleers and Brysbaert, 2010).

Stimuli were recorded by talkers using their phones in .mp4 format, at a sampling frequency of 48,000 Hz. They were then converted to .wav files, segmented in Praat (version 6.2.14; Boersma and Weenink, 2022), and scaled to 70 dB. Experimental stimuli were presented using headphones.

### 2.3 Talkers

Talkers producing the stimuli (*N* = 6; two per location) were native to Seville capital, Seville outskirts, or central Spain. One man and one woman represented each location, the primary criterion being a habitual production of the relevant /s/ variant. Like the participants, talkers spanned a variety of ages (*M* = 47 years) and education levels.

Each talker was sent the list of stimuli and instructed to practice reading all items aloud before recording, later sending the sound files to the experimenter (the first author). Talkers produced the stimuli alone in a quiet location and were told to speak as though they were conversing with their best friend. After review by the experimenter, talkers repeated unclear tokens (e.g., mis-pronounced pseudowords) as requested. Talkers received a virtual gift card of 30 Euro as a token of appreciation.

### 2.4 Procedure

The priming task was completed in OpenSesame (Mathôt et al., 2012). Testing took place in Seville capital, with sessions conducted in Spanish by the experimenter. Participants were told that they would hear six talkers from Spain and to decide whether each item was a real Spanish word.^3^ They were instructed to make a lexical decision as quickly as possible while maintaining accuracy, pressing the ‘s’ key for *sí*, yes, and the ‘n’ key for *no*. If no decision was made within 4,000 ms of the stimulus onset, a new trial began.^4^ There were 1,000 ms between each trial, and no feedback was provided during the experiment.

Participants heard auditory primes in Block 1 followed by auditory targets in Block 2, though the division of the experiment was not made explicit. Each block contained *N* = 360 items, resulting in a long lag between prime and target presentation (i.e., 15–20 minutes). Participants completed the experiment continuously, with an optional break after every 60 trials. The task required approximately 50 minutes to complete, and participants received 30 Euro after the session.

Our experiment featured prime variety as a between-subjects variable (see Sumner and Samuel, 2005, for a similar manipulation). Each participant only heard /s/ primes presented in one variety, resulting in three experimental versions (i.e., Seville primes, Madrid primes, and Afueras primes^5^). Half of the /s/ primes were produced by the man (*N* = 24), and the other half by the woman (*N* = 24). Block 2 included target /s/ words from all six talkers. Prime-target pairs were presented across talker gender to avoid priming from irrelevant acoustic similarities (Church and Schacter, 1994; see also Sumner and Samuel, 2009). Items within blocks were presented in random order, and each *stimulus* (i.e., unique recording) appeared only once. Within Block 2, the full set of critical /s/ words was repeated, along with half of the controls and fillers. The remaining items in Block 2 were new /s/ words, controls, and fillers.

## 3 Results

Statistical analyses were conducted in R Core Team (2021). Participants completed *N* = 720 trials in the form-priming task, yielding *N* = 30,240 responses. We excluded responses from one participant who demonstrated less than 75% accuracy in recognizing real words, as well as four pseudowords with over 50% acceptability (e.g., *cuapa* was acoustically similar to *guapa*, “pretty”). After exclusions, overall accuracy was 87.1%.

To compare accuracy among /s/ words, we ran a logistic regression (ref = control words, Seville). There were no significant differences in accuracy for /s/ words versus controls for Seville or Madrid talkers. However, a significant interaction between Condition and Talker Origin indicated lower accuracy for /s/ words produced by Afueras talkers (*β* = −1.03; *p* < 0.001).

The following analyses concern reaction times (RTs) for /s/ words versus controls (accurate real word trials only; 9.7% of trials were excluded due to inaccuracy). We also excluded responses that fell beyond three SDs from each participant’s average RT (*N* = 147; 1.1% of correct responses). RTs were measured from word offset and log transformed.

### 3.1 Immediate processing

We fit a linear mixed-effects model predicting log RTs as a function of Variant, Talker Gender, and their interaction. Helmert coding was applied to compare (1) the RTs of the non-native variants (i.e., Madrid and Afueras) versus Seville, and (2) RTs for the non-native variants to each other. Participant and Target Word were included as random intercepts. Results from the /s/ word model revealed no significant effects or interactions (*p* > 0.05).

**Figure 1:**
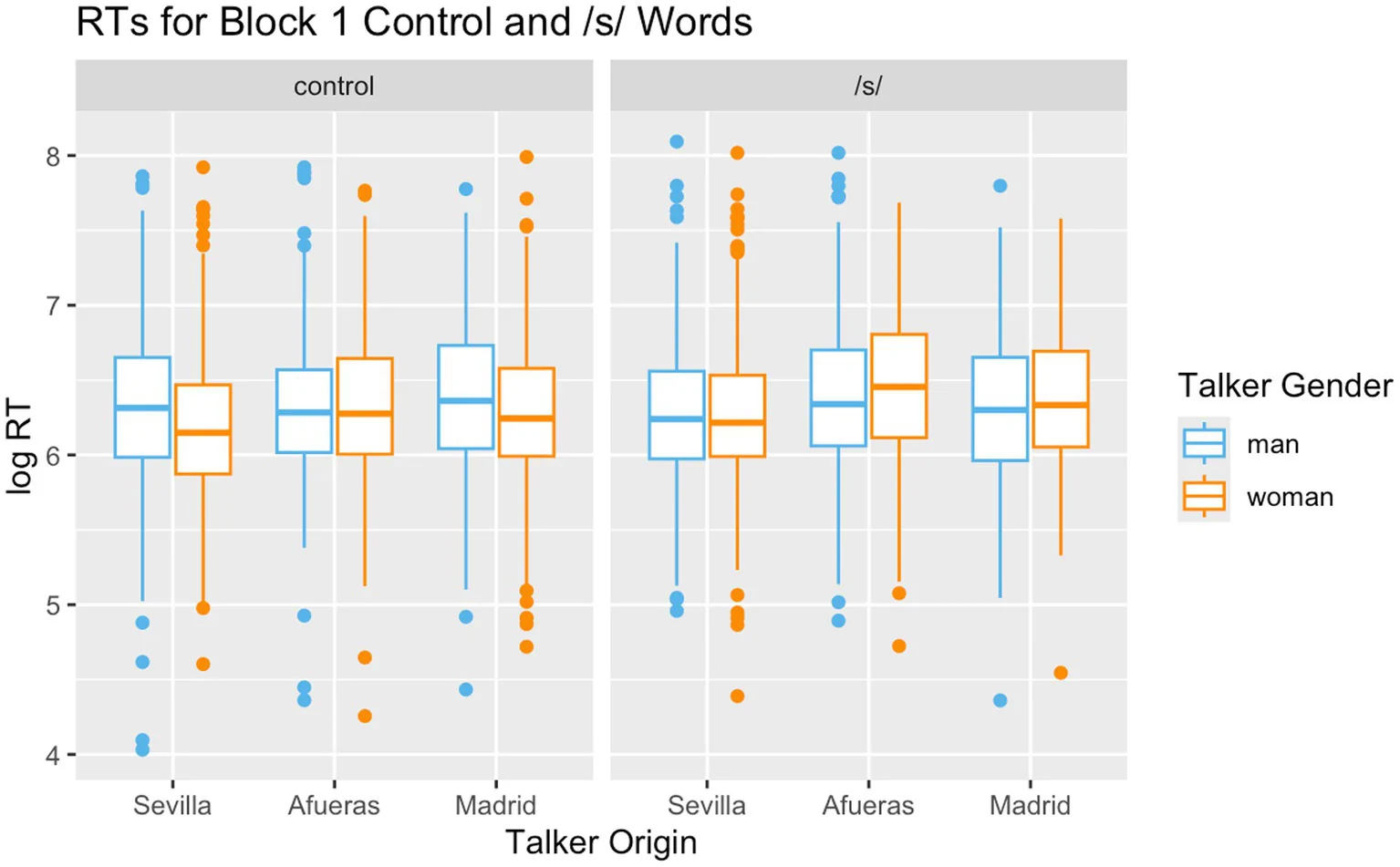
The average reaction times for control and /s/ words in immediate processing. Talker gender is represented by color, with blue for men and orange for women. /S/ words were recognized equivalently, while faster RTs were associated with control words produced by the Seville capital woman.

We also analyzed Block 1 control words, applying the same linear mixed-effects model as above. Results revealed a significant effect for Talker Origin (*β* = −0.02, *p* = 0.036), with controls produced by Seville talkers yielding faster RTs compared to the average of Madrid and Afueras talkers. There were no differences between Madrid and Afueras RTs (*p* > 0.05). A significant interaction between Talker Origin and Gender was observed, with faster RTs for the Seville woman (SW) (*β* = −0.04, *p* = 0.015). *Post-hoc* analysis suggested that SW drove the significant Variety effect, as words produced by the Seville man (SM) were processed significantly slower (*β* = 0.01, *p* = 0.019). Figure 1 represents the faster processing of controls by SW and the equivalent processing of /s/ words.

### 3.2 Long-term processing

**Figure 2:**
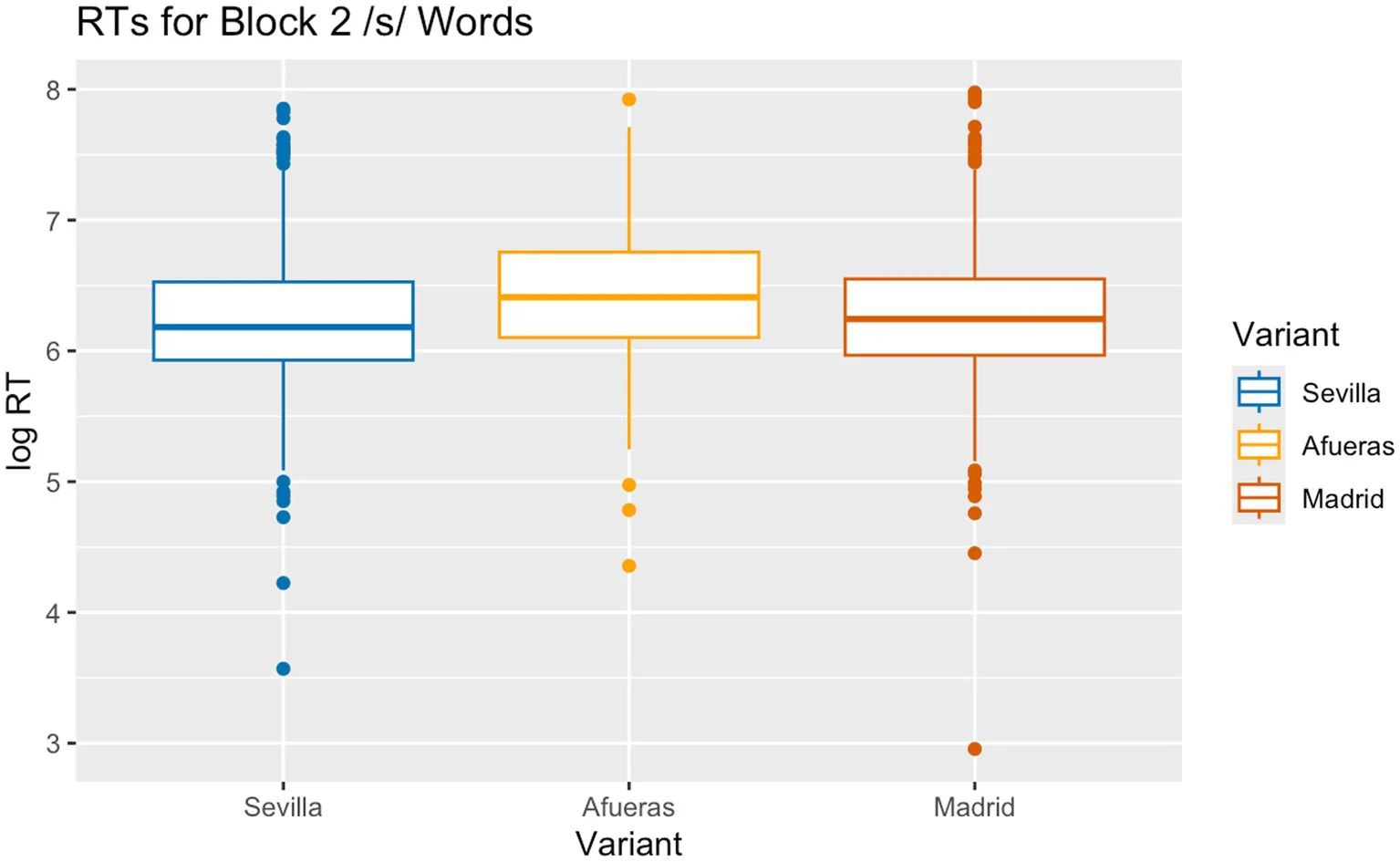
The average reaction times for /s/ words after a long lag of 15–20 minutes from the first presentation. The Madrid and Seville capital variants were recognized faster than the Afueras variant in Block 2.

We analyzed Block 2 RTs to understand whether previous presentation of the target words affected processing. We first considered Block 2 /s/ words, which were all repeated from Block 1 but produced by a different talker, either with the same /s/ variant or a different one. Two tokens were excluded from the analysis due to responses prior to the word offset (*N* = 2/1531 tokens; 0.13% of the data). Helmert coding for Variant and contrast coding for Gender were upheld in Block 2 models, along with the same random intercepts. Table 1 provides average RTs for /s/ words by prime-target pair (see Figure 2).

**Table 1:** Average prime-target RTs for /s/ variant pairs.

Block 1 variant	Mean Block 1 RT	Prime-target pair	Mean Block 2 RT
Seville	608.92 ms	Seville - Seville	549.62 ms
		Seville - Madrid	607.82 ms
		Seville - Afueras	684.18 ms
Madrid	632.87 ms	Madrid - Madrid	588.75 ms
		Madrid - Seville	616.46 ms
		Madrid - Afueras	725.11 ms
Afueras	713.16 ms	Afueras - Afueras	721.65 ms
		Afueras - Seville	621.91 ms
		Afueras - Madrid	629.43 ms

We fit a linear mixed-effects model to log RTs for /s/ words as a function of Prime and Target Variants, along with their interaction. Talker Gender for primes and targets was initially included but neither improved the model nor yielded significance, so it was dropped. The difference between native (i.e., [s̪]) and non-native variants (i.e., [s̺] and [s̪^θ^]) was significant, with participants faster in recognizing /s/ words produced by Seville talkers (*β =* −0.04, *p* < 0.001). Results also revealed a significant effect of Target Variant within the non-native variants, with faster recognition of targets produced by Madrid talkers compared to Afueras (*β* = −0.08, *p* < 0.001). No significant effect of Prime Variant nor interaction between Prime and Target Variants was present. Table 2 depicts the significant effects of Target Variant.

**Table 2:** Summary of Block 1 and 2 RT results.

Stimuli	Block 1	15–20-min lag	Block 2
/s/ words	No effects or interactions.		Effect of target variants: Seville targets faster than combination of Madrid and Afueras (***) Madrid targets faster than Afueras (***) No differences b/w Seville and Madrid
control words	Effect of talker origin, Seville (*) Interaction; effect of talker, SW (*)		Interaction; effect of talker, SW (*)

We conducted a *post-hoc* analysis with Madrid as the reference level to determine whether RTs for Seville and Madrid /s/ words differed from each other. Results indicated no significant differences in RTs (*p* > 0.05), though differences between Madrid and Afueras remained, with slower RTs for the Afueras variant (*p* < 0.01).

Turning to controls, we ran a linear mixed-effects model to analyze Block 2 RTs for repeated control words as a function of Talker Origin, Gender, and their interaction. Similar to results from Block 1 controls, an interaction was observed between Talker Origin and Gender, with significantly faster RTs for words produced by SW (*β* = −0.05, *p* < 0.05).

To test for more global priming, we fit a model comparing repeated versus new Block 2 control words, including fixed effects of Repetition (repeated, new), Talker Origin, Gender, and their interaction. Results revealed no significant repetition effect, with participants no faster in recognizing Block 2 repeated words. However, a significant interaction between Talker Origin and Gender once again demonstrated significantly faster RTs for words produced by SW (*β* = −0.05, *p* = 0.039). Table 2 summarizes results across conditions and blocks.

## 4 Discussion

The current study examined immediate processing and long-term priming of words containing three /s/ variants in Peninsular Spanish, adopting psycholinguistic methodologies. Previous results have shown that in immediate processing, familiar variants can yield equivalent RTs (e.g., Sumner and Samuel, 2009), while a canonical variant tends to be prioritized in memory (e.g., Sumner and Kataoka, 2013). Different patterns may manifest across accuracy and RTs, especially for innovative variants (e.g., López Velarde and Simonet, 2019).

Given previous findings of recognition equivalence in immediate processing by experienced listeners, coupled with Seville listeners’ high familiarity with the /s/ variants, we anticipated that all three variants would be initially recognized similarly. Indeed, immediate processing results suggested that none of the /s/ variants elicited significantly faster RTs. This finding highlights the strong perceptual flexibility of the current listeners and parallels Sumner and Samuel (2009), who found that listeners familiar with NYC English recognized rhotic and non-rhotic variants equivalently in immediate processing.

While RTs demonstrated evidence of recognition equivalence, lower accuracy for the Afueras variant (80.5% correct) suggests some processing difficulty compared to Seville and Madrid variants (89.8% and 91.8% correct, respectively). Considering participants’ familiarity with [s̪^θ^] and prior exposure to the talkers, it is surprising that accuracy rates did not improve throughout the experiment. The word-initial placement of the /s/ variants likely contributed to this result. As no minimal pairs between /s/ and /θ/ were included, if participants initially mapped [s̪^θ^] to /θ/ instead of /s/ (i.e., a result of interdental mixing and increasing presence of /θ/ in Andalusian Spanish) and responded before accessing critical sociodemographic information, the item would likely be perceived as a pseudoword.

Our second research question pertained to the relatively under-researched topic of long-term processing. Given previous findings of memory inequality (e.g., Sumner and Kataoka, 2013), we hypothesized that the Seville and Madrid /s/ variants would both be strongly encoded, the former because it is native, most frequent to the listeners, and holds regional prestige, and the latter due to its canonical status in north-central varieties. While we did not anticipate particularly strong encoding of the Afueras variant, we hypothesized that evidence of encoding could result from its identifiability as a local Andalusian variant and association with regional solidarity. Though a complete examination of the sociolinguistic indexicality of each /s/ variant was outside the scope of the current study, contemporary trends involving the social perception of Andalusian /s/ variants (e.g., Regan, 2022) informed this prediction.

While /s/ words in Block 1 were processed similarly via RTs, differences appeared after a long lag; Block 2 /s/ variants were not all recognized equally by Seville listeners. Significantly faster RTs to targets produced by Seville and Madrid talkers provide preliminary evidence that both [s̪] and [s̺] were strongly encoded. Moreover, there were no differences in processing speed between Seville and Madrid variants. This case of memory inequality, the equal recognition for a less frequent but socially meaningful variant, complements previous findings (e.g., Sumner and Kataoka, 2013).

Despite clear differences between Blocks, we interpret our findings cautiously. Evidence for encoding differences in form-priming paradigms is canonically based on facilitation as a function of particular prime types. In our study, however, no significant effect of Prime Variant nor interaction between Prime and Target Variants were observed. Additionally, there was no repetition effect for repeated controls. While one objective was to include diverse talker voices, this increased variability may have masked clearer priming patterns. Clapp et al. (2023) observed similar findings, in which traditionally stable talker-specificity effects vanished for certain voices upon featuring a broader set of stimuli and listeners.

In form-priming paradigms, a variant typically primes itself more effectively than a different one (i.e., identity priming). As such, we predicted faster RTs for the Seville–Seville condition than Seville–Madrid and Seville–Afueras /s/ pairs. While not statistically significant, this trend was present in the data, with fastest average RTs for the Seville–Seville condition, followed by Madrid–Madrid (see Table 1). More data could clarify these patterns in the future.

Our third research question probed the interaction of social and linguistic information in processing, following recent proposals in the literature (e.g., Clopper, 2021). While we anticipated that participants’ prior completion of a dialect evaluation task could affect processing, we expected these results to influence /s/ words rather than control words. Controls were phonetically and semantically neutral, yet a processing advantage was observed for controls produced by SW, presenting as faster RTs throughout the experiment.

Had we not collected evaluative data, this finding would pose interpretation challenges. However, differences emerged between Seville talkers in the Verbal Guise. Participants were highly accurate in associating SW with Seville capital (90.5% correct), and less accurate for the Seville man (SM) (61.9% correct), with most incorrect answers mistaking him for a resident of central Spain. SW’s voice elicited the highest ratings for solidarity categories (e.g., happy, pretty, proud). Participant feedback in comment boxes also diverged for the Seville talkers. Listeners remarked that SW’s voice was completely identifiable as Sevillian, while forced neutrality was perceived in SM’s voice, reflecting a prominent topic of Andalusian news anchors’ masking of southern traits and adoption of north-central variants (e.g., Santana Marrero, 2022).

Results for /s/ words and controls also patterned differently. No significant talker effects emerged for /s/ words, and all results manifested at the variant level. Why then, did listeners respond to Seville voices similarly for /s/ words but not for controls? We hypothesize that this difference originated in the word-initial position of the /s/ variants, possibly leading to increased or late-resolved uncertainty and reduction of top-down (i.e., context-driven) processing. This interpretation could be supported by the lower accuracy for Afueras /s/ words. Attentional allocation (Clapp and Sumner, 2025) may have also played a role; if Seville participants allocated more attention to /s/ words due to the variability and indexicality of the initial segment, this could have contributed to encoding differences based on talker origin.

While controls did not contain salient phonetic traits, participants’ prior activation of language attitudes may have strengthened the processing of indexical information, resulting in the emergence of top-down effects. This would explain why controls for one specific talker were consistently processed faster, considering participants’ overwhelmingly positive evaluations and strong regional identification of SW. The phonetically neutral controls may also have required fewer cognitive resources to process, freeing up attentional resources for talker-specific qualities.

In summary, our findings suggest differential processing of /s/ words and controls. While /s/ words were recognized equally quickly in immediate processing, Seville and Madrid variants were processed faster and on-par following a long delay between prime-target presentation, indicating that words containing both variants benefited equally from priming. Unexpectedly, control words produced by the Seville woman also yielded processing benefits, likely facilitated by positive social evaluation and regional identifiability. As Seville capital residents are surrounded by an evolving linguistic landscape concerning Andalusian and north-central variants, this social information was available and apparently influential during the recognition task. Limitations to the current study relate to the use of a between-subjects priming manipulation, which possibly made emerging priming patterns harder to detect. Relatedly, due to lower accuracy for Afueras /s/ words, these are less represented in the data. Including more participants in future work can help resolve these issues. Subsequent studies can also consider inter-talker consistency measures to ensure better comparability of stimuli across variants.

To conclude, we provide evidence from Peninsular Spanish that what is being said and by whom are not separate entities, but rather deeply connected. Our task combination leveraged this intersection, highlighting the importance of considering participants’ attitudes towards the specific variants, varieties, and talkers appearing in priming tasks. Future work will do well to consider non-English variants and their social meanings in service of advancing our understanding of spoken word recognition.

## Data Availability

The raw data supporting the conclusions of this article will be made available by the authors, without undue reservation.

## Footnotes

1 *Seseo* and *ceceo* are phonemic mergers. The /s/ variants are their phonetic correlates, with [s̪] produced in Sevillian *seseo* and [s̪^θ^] in *ceceo*.

2 One participant reported moving to Seville capital when they were 1 month old.

3 Participants responded according to whether the word held meaning to them, regardless of dictionary status.

4 A deadline of 3,000 ms from word onset is fairly standard in studies of English auditory word recognition (e.g., Tucker et al., 2019). We chose 4,000 ms because our Spanish stimuli are disyllabic, slightly longer than most English stimuli, and our participants represent a more varied population considering age and socioeconomic status. A response window of 4,000 ms has been used in other lexical decision tasks featuring Spanish disyllabic stimuli (e.g., Daidone and Darcy, 2021).

5 Talker origins will be referenced as Seville (i.e., Seville capital), Madrid (i.e., central Spain), and Afueras (i.e., *las afueras de Sevilla*, ‘Seville outskirts’).

## Supplementary material

The Supplementary material can be found online at: https://www.frontiersin.org/articles/10.3389/fpsyg.2026.1832323/full#supplementary-material
