# Supplementary material for: When control words lose control: emergence of top-down processing effects in Spanish spoken word recognition
Source: Front Psychol. 2026 Sep 3;17:1832323. doi: 10.3389/fpsyg.2026.1832323 (PMC13599132; doi:10.3389/fpsyg.2026.1832323)
Supplement: Supplementary file 1 [file Data_Sheet_1.PDF]

These materials include:

A. All items from the form-priming task and their conditions.
B. Model outputs from form-priming task.

A. All stimuli appearing in form-priming task; items in each table ordered from least – most frequent

/s/ words (N = 48) **control words (N = 48)** **new controls (N = 24)**

Word	Translation	Word	Translation	Word	Translation
sarro	plaque (dental)	*cacto*	cactus	tromba	whirlwind
sorna	sarcasm	*diabla*	devil	naipe	card
sardo	Sardinian	*tilde*	accent mark	grapa	staple
surco	groove	*miga*	crumb	orca	killer whale
seto	hedge	*leño*	log	mote	nickname
salvia	sage	*trufa*	truffle	tecla	keyboard key
sigma	sigma (number)	*gamba*	prawn	podio	podium
salmo	psalm	*pana*	corduroy	cono	cone
soto	thicket	*cuña*	crib	aula	classroom
sarta	string	*bucle*	loop	piña	pineapple
silba	whistle	*fierro*	iron	bota	boot
silo	silo	*cuenco*	bowl	ruina	ruin
saldo	balance	*helio*	helium	falda	skirt
sodio	sodium	*viudo*	widow	beca	scholarship
sauna	sauna	*lomo*	loin	nido	nest
senda	track	*caldo*	broth	horno	oven
sorbo	sip	*peine*	comb	barba	beard
simio	ape	*hongo*	mushroom	fiebre	fever
soplo	breath	*ancla*	anchor	venta	sale
secta	sect	*loro*	parrot	lengua	language
sable	saber	*himno*	hymn	banda	band
sabio	sage; intellect	*finca*	farm	foto	picture
sapo	toad	*dato*	fact	fuego	fire
suegra	mother-in-law	*menta*	mint	tierra	land
sonda	probe	*nieta*	granddaughter		
suero	serum	*gripe*	flu		
soda	soda water	*mito*	myth		
sexto	sixth	*diente*	tooth		

soga	rope	*pila*	battery
signo	sign	*nube*	cloud
suma	sum	*feria*	fair
selva	jungle	*burro*	donkey
sueldo	salary	*tarta*	cake
salto	jump; skip	*palo*	stick
salsa	sauce	*templo*	temple
sombra	shadow	*moda*	fashion
sopa	soup	*ritmo*	rhythm
saco	sack; bag	*plata*	silver
siglo	century	*barrio*	neighborhood
serie	series	*polvo*	dust
santo	saint	*puente*	bridge
sala	room	*luna*	moon
suelo	floor	*norte*	north
sexo	sex	*tienda*	store
sueño	dream	*grupo*	group
sitio	place	*libro*	book
sangre	blood	*cara*	face
suerte	luck	*agua*	water

Real filler words (N = 84) **New filler words (N = 44)**

**Word**	**Translation**	**Word**	**Translation**
yuca	yuca	ubres	utters
churros	churros	caqui	khacki
mimbre	wicker	mella	dent
equis	x	cóndor	condor
facha	look; figure	ostra	oyster
plagio	plagiarism	franja	fringe
yema	yoke	rasgo	trait
rizo	curl	calcio	calcium
dorso	back	pera	pear
gruta	grotto	tapas	tapas
guiño	wink	soja	soy
fósil	fossil	mugre	dirt
ocio	leisure	charco	puddle
bache	pothole	cejas	eyebrows
ámbar	amber	ciervo	deer

lienzo	canvas	zumo	juice
jerga	slang	monja	monk
prendas	garments	frenos	brakes
chisme	gossip	talla	size
mangas	sleeves	sello	stamp
plancha	iron	susto	scare
rabo	tail	pesca	fishing
mármol	marble	hombros	shoulders
fresa	strawberry	brindis	cheers
sobras	leftovers	medias	tights
jarra	jar	lujo	luxury
olla	pot	jefa	boss
cancha	court	móvil	cell phone
ajo	garlic	bruja	witch
frutas	fruits	lago	lake
pausa	pause	techo	roof
bicho	beatle	prensa	press
zorro	fox	leche	milk
siesta	nap	beso	kiss
sierra	mountain range	precio	price
raya	stripe	ganas	desire
grasa	fat	isla	island
queja	complaint	zona	zone
trenes	trains	radio	radio
vidrio	glass	centro	center
copas	cups	pelo	hair
huella	fingerprint	coche	car
mancha	stain	ojos	eyes
postre	dessert	muerte	death
muebles	furniture		
letras	letters		
hoja	leaf		
risa	laugh		
frase	phrase		
barcos	boats		
cuadro	painting		
joyas	jewels		
plaza	plaza		

túnel	tunnel
seres	beings
fecha	date
gira	tour
notas	grades
granja	farm
queso	cheese
lluvia	rain
ángel	angel
costa	coast
silla	chair
ruido	noise
brazos	arms
hielo	ice
trampa	trick
flores	flowers
fondo	bottom
vuelo	flight
reina	queen
cárcel	jail
alma	soul
mesa	table
traje	suit
fuerza	force
trato	treaty
cielo	sky
falta	lack
hijos	kids
horas	hours
miedo	fear
clase	class

Pseudoword controls (N = 48) **New pseudo controls (N = 24)**

Pseudo	Derived from	Translation	Pseudo	Derived from	Translation
conlo	calco	tracing	bestro	bistro	bistro
galmo	galgo	greyhound	dedro	cedro	cedar tree
lirra	birra	beer	fucto	ducto	pipeline

fialo	fauno	faun
guna	duna	dune
mada	cata	tasting
truña	trufa	truffle
fueto	dueto	duet
ririo	lirio	lily
vermo	verbo	verb
linte	tinte	dye
meño	moño	bun
bloro	cloro	chlorine
bimo	lino	linen
darco	dardo	dart
rego	remo	oar
plauta	flauta	flute
fuende	duende	elf
marno	mango	mango
dibra	fibra	fiber
truelo	trueno	thunder
nuzo	nudo	knot
plemo	plomo	lead
gaca	hada	fairy
milta	multa	fine
huedo	hueco	hole
blobo	globo	globe
cagra	cabra	goat
niedra	niebla	fog
glama	drama	drama
dena	vela	candle
mirte	monte	mountain
ruema	rueda	wheel
flima	clima	climate
panio	patio	patio
pima	mina	mine
raca	pata	paw
meblo	metro	metro
dumo	humo	smoke
clape	clave	key
rorre	torre	tower
fiario	diario	diary

barlo	bardo	bard
belmo	bombo	drum
afre	acre	acre
dorro	forro	cover
euna	aura	aura
crote	brote	outbreak
flera	flora	vegetation
firo	faro	lighthouse
trimo	trigo	wheat
papria	patria	homeland
crito	grito	shout
binta	quinta	country house
cuepa	cueva	cave
rigre	tigre	tiger
houda	deuda	debt
rura	ruta	route
retla	regla	rule
malpa	mapa	map
fample	hambre	hunger
nepia	novia	bride; girlfriend
merro	perro	dog

piegra	piedra	stone
obla	obra	work
iare	aire	air
reba	ropa	clothing
puebro	pueblo	town
vuerra	guerra	war

Pseudoword fillers (*N* = 132) **New pseudo fillers (*N* = 66)**

Pseudo	Derived from	Translation	Pseudo	Derived from	Translation
plimas	plisas	Breezes	lostre	lustre	gloss
carfe	canje	exchange	sirra	mirra	myrrh
ajia	apio	celery	calpa	caspa	dandruff
euge	auge	boom	fiosas	diosas	goddesses
fanzas	danzas	dances	resca	rosca	thread
gesco	vasco	Basque	rerno	termo	thermos
hichas	hachas	axes	paitas	pautas	guidelines
cengo	censo	census	noya	boya	buoy
corjus	corpus	corpus	pulma	pulga	flea
lugro	lucro	profit	febia	fobia	phobia
duerta	huerta	orchard	diedra	hiedra	ivy
dallos	gallos	rooster	muema	muela	molar
dactros	filtros	filters	akras	atlas	atlas
trempa	trompa	horn	letas	setas	mushrooms
samla	samba	samba	baichas	bayas	berries
banfo	banjo	banjo	arja	arpa	harp
péster	póster	poster	verpa	verja	gate
mebra	cebra	zebra	balna	balsa	raft
penza	pinza	clip	bunda	funda	cover
alclo	astro	star	liegre	liebre	hare
gada	gama	scale	trurra	trucha	trout
frimas	primas	girl cousins	chijo	chivo	goat
lollo	bollo	scone	bomas	bocas	mouths
crapta	cripta	crypt	colso	colmo	height
custe	coste	cost	ganro	ganso	goose
mafo	mazo	gavel	custa	cesta	basket
lonte	lente	lens	flardo	flanco	flank
jaunas	jaulas	cages	tarno	tango	tango

bilto	bulto	package	darras	garras	claws
palgas	palmas	palms	viendre	vientre	stomach
sodia	socia	member	lorta	torta	cake
musno	muslo	thigh	lluño	puño	fist
groche	broche	brooch	mundes	mundos	worlds
balne	balde	pail	lópiz	lápiz	pencil
trispas	chispas	sparks	refto	texto	text
diso	filo	edge	rarcho	rancho	ranch
yiso	yeso	cast	huinza	fianza	deposit
vimio	vicio	vice	labla	lata	tin
licas	ligas	leagues	mava	masa	dough
flantas	llantas	tires	cafas	papas	potatoes
rospa	rampa	ramp	maos	caos	chaos
rogre	roble	oak	goco	voto	vote
suemo	sueco	Swedish	ulas	alas	wings
legros	logros	achievements	ausco	asco	disgust
llotos	motos	motorcycles	charga	charla	chat
asla	asma	asthma	nunta	junta	board
cisle	cisne	swan	guka	guía	guide
resis	tesis	thesis	pados	pasos	steps
fuepos	huevos	eggs	piaza	pieza	piece
dasto	gasto	expense	clanta	planta	plant
ucas	uvas	grapes	bistia	bestia	beast
taga	tasa	rate	nieje	nieve	snow
yona	yoga	yoga	ciercia	ciencia	science
pemos	pesos	weights	duente	fuente	fountain
racas	ramas	branches	hoches	hechos	facts
rares	mares	seas	pamas	casas	houses
folo	velo	veil	carsa	carga	burden
nuemes	nueces	nuts	numbros	nombres	names
rigles	rifles	rifles	manlla	marcha	progress
yete	yate	yacht	tuelvos	tiempos	times
chicre	chicle	gum	pusta	pista	clue
floque	bloque	block	bame	base	base
chiunfo	triunfo	triumph	olo	oro	gold
álsum	álbum	album	crisa	prisa	hurry
munos	muros	walls	campue	cambio	change
flaga	plaga	plague	argas	armas	weapons
blecha	flecha	arrow			

nerma	norma	norm
juedos	jueces	judges
misca	mosca	fly
fueños	dueños	owners
bafos	baños	baths
rinta	renta	rent
fruce	cruce	cross
ogos	osos	bears
cagles	cables	cables
chostes	chistes	jokes
mubo	cubo	cube
mele	coro	choir
pento	parto	birth
pamos	pavos	turkeys
vieta	dieta	diet
duelga	huelga	strike
mialo	cuero	leather
dacho	fallo	failure
gisto	gesto	gesture
marde	marte	Mars
melos	celos	jealousy
loda	gota	drop
mecra	mezcla	mix
vama	fama	fame
trolo	trono	throne
umas	uñas	nails
vomes	voces	voices
nersias	nervios	nerves
gaxas	gafas	glasses
nalle	valle	valley
ranque	tanque	tank
tama	lana	wool
tíes	tíos	uncles
compnas	compras	shopping
tabios	labios	lips
fúnbol	fútbol	soccer
firga	firma	signature
juemes	juegos	games
porbo	pollo	chicken

masia	magia	magic
líser	líder	leader
pade	pago	payment
carres	calles	streets
cergo	cerdo	pig
pluya	playa	beach
lurra	lucha	fight
órbol	árbol	tree
golre	golpe	hit
blogas	drogas	drugs
flave	llave	key
cricen	crimen	crime
llajas	llamas	flames
pono	modo	mode
grueba	prueba	quiz
giaje	viaje	trip
erden	orden	order
cunsto	cuarto	room
maba	cama	bed
mosio	medio	medium
cuapa	culpa	fault
diasta	fiesta	party
viepta	vuelta	return
cuerjo	cuerpo	body
medes	meses	months
nochos	noches	nights

B. Model outputs for Block 1 & 2 RTs

Block 1 /s/ words

```
Linear mixed model fit by REML. t-tests use Satterthwaite's method [lmerModLmerTest
]
Formula: log(Block1_rt_adj) ~ (1 | Target) + (1 | subject_nr) + Variety +
    Gender + Variety * Gender
   Data: Block1_s

REML criterion at convergence: 2090.1

Scaled residuals:
    Min      1Q  Median      3Q     Max
-4.1011 -0.6174 -0.1034  0.5592  4.2425

Random effects:
 Groups     Name        Variance Std.Dev.
 Target     (Intercept) 0.01951  0.1397
 subject_nr (Intercept) 0.05588  0.2364
 Residual               0.18048  0.4248
Number of obs: 1675, groups:  Target, 48; subject_nr, 41

Fixed effects:
                       Estimate Std. Error        df t value Pr(>|t|)
(Intercept)           6.346e+00  4.883e-02  6.892e+01 129.971   <2e-16 ***
Variety1             -4.560e-02  4.886e-02  4.310e+01  -0.933    0.356
Variety2             -2.659e-02  2.784e-02  4.299e+01  -0.955    0.345
Genderwoman           5.364e-02  4.561e-02  4.303e+01   1.176    0.246
Variety1:Genderwoman -6.879e-03  2.633e-02  1.589e+03  -0.261    0.794
Variety2:Genderwoman -1.815e-02  1.453e-02  1.588e+03  -1.249    0.212
---
Signif. codes:  0 ‘***’ 0.001 ‘**’ 0.01 ‘*’ 0.05 ‘.’ 0.1 ‘ ’ 1

Correlation of Fixed Effects:
            (Intr) Varty1 Varty2 Gndrwm Vrt1:G
Variety1    -0.026
Variety2    -0.015  0.023
Genderwoman -0.458  0.004  0.003
Vrty1:Gndrw  0.007 -0.244 -0.006 -0.042
Vrty2:Gndrw  0.005 -0.007 -0.250 -0.032  0.066
```

Block 1 controls

```
Linear mixed model fit by REML. t-tests use Satterthwaite's method [lmerModLmerTest
]
Formula: log(RT) ~ (1 | Target) + (1 | subject_nr) + Variety + Gender +
    Variety * Gender
   Data: Block1_controls

REML criterion at convergence: 2323.3

Scaled residuals:
    Min      1Q  Median      3Q     Max
-4.2018 -0.6101 -0.0924  0.5368  3.8077

Random effects:
 Groups     Name        Variance Std.Dev.
 Target     (Intercept) 0.02231  0.1494
 subject_nr (Intercept) 0.05188  0.2278
 Residual               0.19002  0.4359
Number of obs: 1792, groups:  Target, 48; subject_nr, 41

Fixed effects:
                       Estimate Std. Error         df t value Pr(>|t|)
(Intercept)           6.344e+00  4.910e-02  7.586e+01 129.191   <2e-16 ***
Variety1              2.698e-02  1.787e-02  1.706e+03   1.510   0.1312
Variety2              2.566e-03  1.043e-02  1.703e+03   0.246   0.8057
Genderwoman          -6.246e-02  4.786e-02  4.415e+01  -1.305   0.1986
Variety1:Genderwoman -4.194e-02  2.539e-02  1.705e+03  -1.652   0.0988 .
Variety2:Genderwoman -3.569e-02  1.458e-02  1.702e+03  -2.448   0.0145 *
---
Signif. codes:  0 ‘***’ 0.001 ‘**’ 0.01 ‘*’ 0.05 ‘.’ 0.1 ‘ ’ 1

Correlation of Fixed Effects:
            (Intr) Varty1 Varty2 Gndrwm Vrt1:G
Variety1     0.006
Variety2     0.006 -0.014
Genderwoman -0.487 -0.007 -0.006
Vrty1:Gndrw -0.005 -0.704  0.010  0.001
Vrty2:Gndrw -0.004  0.010 -0.715 -0.003 -0.001
```

Block 2 /s/ words

```
Linear mixed model fit by REML. t-tests use Satterthwaite's method [lmerModLmerTest
]
Formula: log(Block2_rt_adj) ~ (1 | Target) + (1 | subject_nr) + Variety.x +
    Variety.y + Gender.x + Gender.y + Variety.x * Variety.y
   Data: Block2_s

REML criterion at convergence: 2138.3

Scaled residuals:
    Min      1Q  Median      3Q     Max
-5.7038 -0.6181 -0.0823  0.5298  3.6961

Random effects:
 Groups     Name        Variance Std.Dev.
 Target     (Intercept) 0.01122  0.1059
 subject_nr (Intercept) 0.05188  0.2278
 Residual               0.20899  0.4572
Number of obs: 1529, groups:  Target, 48; subject_nr, 41

Fixed effects:
                       Estimate Std. Error         df t value Pr(>|t|)
(Intercept)           6.298e+00  4.476e-02  6.176e+01 140.694  < 2e-16 ***
Variety.x1            1.353e-03  4.628e-02  3.810e+01   0.029   0.9768
Variety.x2           -1.586e-02  2.635e-02  3.790e+01  -0.602   0.5508
Variety.y1           -8.162e-02  1.620e-02  9.780e+02  -5.040 5.55e-07 ***
Variety.y2           -4.033e-02  9.130e-03  1.040e+03  -4.417 1.10e-05 ***
Gender.xmujer         7.469e-02  3.885e-02  4.455e+01   1.923   0.0609 .
Variety.x1:Variety.y1 -6.834e-03  2.001e-02  9.060e+02  -0.342   0.7328
Variety.x2:Variety.y1 -6.010e-03  1.225e-02  4.519e+02  -0.491   0.6238
Variety.x1:Variety.y2  3.470e-03  1.187e-02  6.778e+02   0.292   0.7701
Variety.x2:Variety.y2 -7.423e-03  6.837e-03  4.615e+02  -1.086   0.2782
---
Signif. codes:  0 ‘***’ 0.001 ‘**’ 0.01 ‘*’ 0.05 ‘.’ 0.1 ‘ ’ 1

Correlation of Fixed Effects:
            (Intr) Vrty.x1 Vrty.x2 Vrty.y1 Vrty.y2 Gndr.x V.1:V.1 V.2:V.1 V.1:V.2
Variety.x1  -0.022
Variety.x2  -0.013  0.024
Variety.y1  -0.010  0.000  -0.011
Variety.y2  -0.016 -0.009  -0.011   0.112
Gender.xmjr -0.423 -0.013  -0.009   0.002   0.027
Vrty.x1:V.1 -0.009 -0.006  -0.003  -0.036   0.008   0.018
Vrty.x2:V.1 -0.012  0.003  -0.019  -0.042   0.076   0.012 -0.019
Vrty.x1:V.2 -0.005  0.000   0.008   0.003  -0.023  -0.004  0.041  -0.156
Vrty.x2:V.2 -0.006  0.003  -0.013   0.038  -0.061   0.005  0.154  -0.098   0.138
fit warnings:
fixed-effect model matrix is rank deficient so dropping 1 column / coefficient
```

Block 2 repeated controls

```
Linear mixed model fit by REML. t-tests use Satterthwaite's method [lmerModLmerTest
]
Formula: log(Block2_rt_adj) ~ (1 | Target) + (1 | subject_nr) + Variety.y +
    Gender.y + Variety.y * Gender.y
   Data: Block2_repcontrols

REML criterion at convergence: 1170.1

Scaled residuals:
    Min      1Q  Median      3Q     Max
-3.7830 -0.6110 -0.0752  0.5556  3.3428

Random effects:
 Groups     Name        Variance Std.Dev.
 subject_nr (Intercept) 0.08984  0.2997
 Target     (Intercept) 0.01365  0.1168
 Residual               0.21233  0.4608
Number of obs: 798, groups:  subject_nr, 41; Target, 24

Fixed effects:
                     Estimate Std. Error        df t value Pr(>|t|)
(Intercept)           6.32671    0.05514  49.44514 114.733   <2e-16 ***
Variety.y1            0.01108    0.02025 739.00484   0.547   0.5845
Variety.y2           -0.02186    0.01164 736.71461  -1.879   0.0606 .
Gender.y1            -0.05006    0.05818  19.49023  -0.860   0.4000
Variety.y1:Gender.y1 -0.06862    0.04041 738.27306  -1.698   0.0899 .
Variety.y2:Gender.y1 -0.05072    0.02332 737.61634  -2.175   0.0299 *
---
Signif. codes:  0 ‘***’ 0.001 ‘**’ 0.01 ‘*’ 0.05 ‘.’ 0.1 ‘ ’ 1

Correlation of Fixed Effects:
            (Intr) Vrty.1 Vrty.2 Gndr.1 V.1:G.
Variety.y1   0.005
Variety.y2   0.000 -0.008
Gender.y1    0.006 -0.013 -0.031
Vrty.y1:G.1 -0.008  0.058  0.016  0.009
Vrty.y2:G.1 -0.016  0.023 -0.028  0.000 -0.003
```

Block 2 new controls vs. repeated controls

```
Linear mixed model fit by REML. t-tests use Satterthwaite's method [lmerModLmerTest
]
Formula: log(Block2_rt_adj) ~ (1 | Target) + (1 | subject_nr) + Repetition +
    Variety + Gender + Repetition * Variety * Gender
   Data: Block2_rc_repnew

REML criterion at convergence: 2460.4

Scaled residuals:
    Min      1Q  Median      3Q     Max
-4.1272 -0.6349 -0.0977  0.5425  3.8310

Random effects:
 Groups     Name        Variance Std.Dev.
 Target     (Intercept) 0.01411  0.1188
 subject_nr (Intercept) 0.07991  0.2827
 Residual               0.22627  0.4757
Number of obs: 1660, groups:  Target, 48; subject_nr, 41

Fixed effects:
                                Estimate Std. Error         df t value Pr(>|t|)
(Intercept)                      6.32387    0.05323   63.28499 118.807   <2e-16
Repetitionnew                    0.05452    0.04169   40.84941   1.308   0.1984
Variety1                         0.01385    0.02084 1579.64558   0.664   0.5065
Variety2                        -0.02198    0.01199 1576.84237  -1.833   0.0670
Gender1                         -0.05179    0.05941   41.84105  -0.872   0.3884
Repetitionnew:Variety1          -0.03149    0.02902 1580.20067  -1.085   0.2782
Repetitionnew:Variety2          -0.01119    0.01657 1575.89382  -0.675   0.4995
Repetitionnew:Gender1            0.04534    0.08336   40.79857   0.544   0.5894
Variety1:Gender1                -0.06736    0.04164 1579.28370  -1.618   0.1059
Variety2:Gender1                -0.04966    0.02400 1577.47779  -2.069   0.0387
Repetitionnew:Variety1:Gender1   0.01843    0.05799 1579.82769   0.318   0.7507
Repetitionnew:Variety2:Gender1   0.02176    0.03320 1577.70560   0.656   0.5122

(Intercept)                    ***
Repetitionnew
Variety1
Variety2                       .
Gender1
Repetitionnew:Variety1
Repetitionnew:Variety2
Repetitionnew:Gender1
Variety1:Gender1
Variety2:Gender1               *
Repetitionnew:Variety1:Gender1
Repetitionnew:Variety2:Gender1
---
Signif. codes:  0 '***' 0.001 '**' 0.01 '*' 0.05 '.' 0.1 ' ' 1
```
